# Unintentional Staining of the Anterior Vitreous with Trypan Blue During Cataract Surgery

**DOI:** 10.4274/tjo.galenos.2020.94580

**Published:** 2020-10-30

**Authors:** Özcan Rasim Kayıkçıoğlu, Hüseyin Mayalı, Suzan Doğruya, Şenay Alp, Aydın Alper Yılmazlar, Emin Kurt

**Affiliations:** 1 Celal Bayar University Faculty of Medicine, Department of Ophthalmology, Manisa, Turkey; 2 Uşak University Training and Research Hospital, Clinic of Ophthalmology, Uşak, Turkey; 3 Adıyaman Training and Researh Hospital, Clinic of Ophthalmology, Adıyaman, Turkey; 4 Aydın State Hospital, Aydın, Turkey

**Keywords:** Trypan blue, phacoemulsification, vitreous

## Abstract

During phacoemulsification and intraocular lens (IOL) implantation surgery, the trypan blue dye used to stain the anterior capsule passed into vitreous cavity and stained the anterior capsule and anterior vitreous in 6 patients. There was history of trauma in 2 patients, uveitis in 1 patient, mature cataract in 1 patient, and no risk factors in the other patients. IOL was implanted in-the-bag without problem in 5 patients. In the patient with iris and zonular defects due to trauma, a sutured IOL was implanted in the same session. The migration of trypan blue into the vitreous cavity through damaged or intact lens zonules is a rare but important complication that makes subsequent surgical steps substantially more difficult.

## Introduction

Trypan blue is a dye that facilitates anterior lens capsule visualization and capsule manipulation during cataract surgery.^1,2^ Trypan blue staining of the posterior capsule and anterior vitreous is uncommon; reports in the literature indicate that it may occur in eyes with ocular trauma, history of ocular surgery, or pseudoexfoliation.^3,4,5,6,7^ Migration of dye to the posterior chamber and vitreous cavity through areas of zonular weakness has been implicated as the probable cause of anterior vitreous and posterior capsule staining.

The aim of this study was to present cases in which complete loss of the red reflex was observed due to trypan blue migrating to the posterior chamber and staining the posterior capsule and anterior vitreous during cataract surgery, and to evaluate this rare clinical picture.

## Case Report

Six cases in which trypan blue stained the posterior capsule and anterior vitreous during cataract surgery performed in the ophthalmology department of Manisa Celal Bayar University Faculty of Medicine are presented (Table 1). Patient characteristics, surgical difficulties encountered, and features that facilitated the migration of trypan blue to the anterior vitreous are presented.

### Case 1

**Figure 1:**
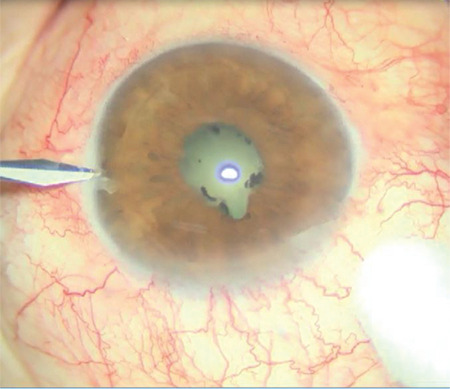
Posterior synechia, small pupil, and cataract reflex are observed in the peroperative initial appearance of our uveitic patient (patient 1)

**Figure 2:**
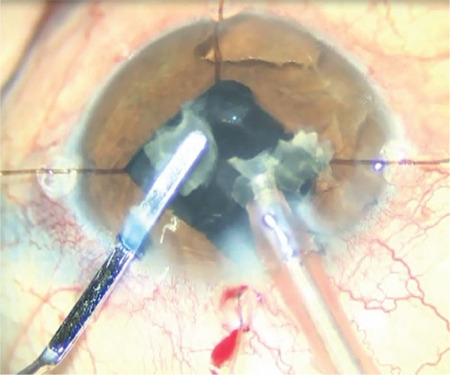
Disappearance of the fundus reflex is observed during phacoemulsification with iris retractors

**Figure 3:**
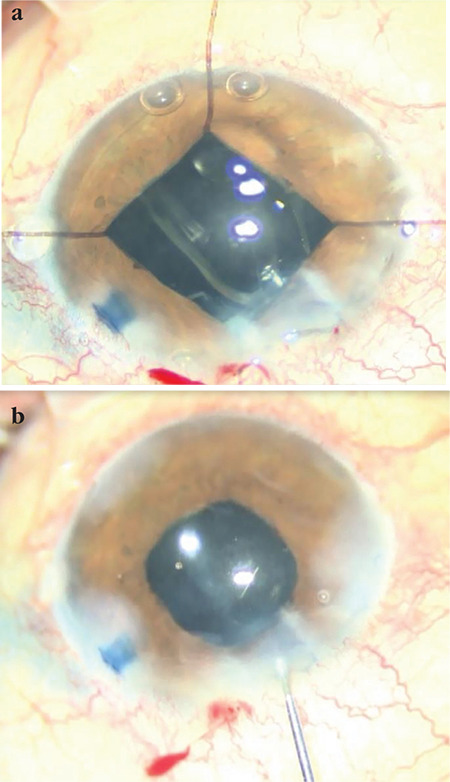
Intraocular lens placement (a) and appearance at the end of the operation (b)

A 62-year-old woman had preoperative visual acuity of counting fingers from 30 cm, posterior synechia secondary to previous uveitic episodes, and nuclear cataract in the left eye (Figure 1). During cataract surgery, the anterior chamber was filled with dispersive-cohesive viscoelastic and iris retractors were used to provide pupil dilation and the capsule was stained with 0.06% trypan blue under viscoelastic. Anterior capsulorhexis was performed. No signs of zonular defect were encountered during the operative stages. While emulsifying the nucleus fragments, complete loss of the red reflex was observed (Figure 2). No anterior chamber shallowness or ocular rigidity that would suggest suprachoroidal hemorrhage was detected. Cortex aspiration was completed with the irrigation/aspiration cannula and a foldable IOL was implanted in the capsular sac (Figure 3a, b). The posterior capsule and anterior vitreous staining observed on biomicroscopic examination on postoperative day 1 decreased on day 2 and disappeared completely in the following days.

### Case 2

**Figure 4:**
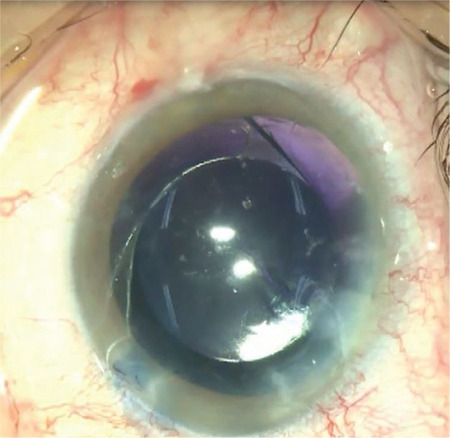
Trypan blue staining of the posterior capsule and anterior vitreous is observed (patient 2

A 39-year-old man had visual acuity of 0.2 in his right eye and extensive corticonuclear cataract on anterior segment examination. His history included information about ocular trauma. During cataract surgery, 0.06% trypan blue was administered to the anterior chamber under air and irrigated, and the anterior chamber was filled with a dispersive-cohesive viscoelastic substance. The red reflex was not observed during nucleus phacoemulsification, but the operative stages were completed without any problems. The IOL was inserted into the capsular sac without difficulty (Figure 4). The trypan blue staining disappeared with a few days after the operation without causing any surgical complications or problems in postoperative follow-up.

**Figure 5:**
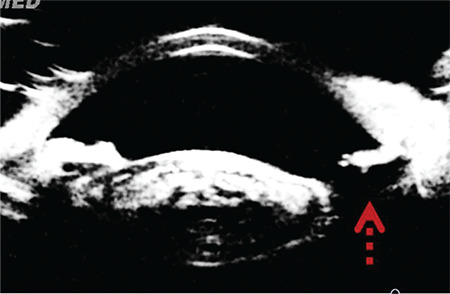
Ultrasound biomicroscopy reveals iris and zonular defects and lens opacity (patient 5). The red arrow indicates the area of zonular damage

Of the other cases, only the patient with ocular trauma (patient 5) exhibited atrophy in the upper half of the iris and zonular defect detected by ultrasound biomicroscopic imaging (UBM) in the preoperative evaluation performed due to the trauma (Figure 5). Trypan blue staining of the posterior capsule was attributed to the zonular defect in this patient.

In the uveitic patient, trypan blue staining was performed under viscoelastic. In the other patients, trypan blue was administered to the anterior chamber under air, irrigated, and the anterior chamber was filled with dispersive-cohesive viscoelastic (DisCoVisc OVD, Alcon, TX, USA). The patients showed no signs of phacodonesis, iridodonesis, or pseudoexfoliation.

In all patients, red reflex was absent on biomicroscopic examination on the first postoperative day, but the staining cleared rapidly. One week later, there was no sign of staining in the posterior segment and the retina could be evaluated easily. Postoperative UBM in 3 patients (patients 1-3) revealed no evidence of zonular damage.

## Discussion

In case reports of inadvertent posterior capsule staining in the literature, this phenomenon occured in eyes with pseudoexfoliation, ocular trauma, or ocular surgery history, and it was suggested that zonular weakness may be responsible.^3,4,5,6,7^ However, two published case reports demonstrated that trypan blue staining of the posterior capsule is also possible even in eyes without history of surgery or trauma; regarding the etiology, the administration of additional viscoelastic was posited to increase intraocular pressure and force the posterior migration of the dye through intact zonules.^8,9^ In addition, the intraoperative use of iris retractors to dilate the pupil or stabilize a floppy iris may cause inadvertent staining of the posterior capsule. The authors proposed that this was because iris retractors lift the iris, thereby facilitating the passage of trypan blue through the intact zonules to the posterior chamber.^10^ Anterior vitreous staining with trypan blue may occur in cases without pre- or intraoperative zonular dialysis but with peripheral iridotomy or conditions that may damage the anterior hyaloid surface or zonular apparatus.^11^

In our patient with uveitis and synechiae (patient 1), the use of an iris retractor due to small pupil may have facilitated trypan blue migration to the posterior chamber. In our patients with history of trauma (patients 2 and 5), trypan blue staining of the anterior vitreous occured due to zonular damage. The absence of known risk factors in the other two patients (patients 3 and 4) suggests that the surgical method may also have caused dye migration through the intact zonules. In our patient with mature cataract (patient 6), although we noted no risk factors during surgery, staining of the anterior vitreous suggests abnormality in the zonular apparatus. None of our patients had a history of using prostate medication, so our patient group did not include iris atrophy and floppy iris.

Different methods have been recommended for more effective trypan blue staining of the anterior capsule with less toxicity to the endothelium. The purpose of staining under air is to protect the corneal endothelium. Alternative methods have been developed, such as staining the anterior capsule with trypan blue under viscoelastic, attaching iris retractors parallel to the iris plane without allowing iris elevation, injecting viscoelastic to the peripheral iridolenticular area to act as a barrier, and using a mixture of viscoelastic and trypan blue.^5,7,12,13,14^

The main purpose of staining under air is to prevent dye contact with the corneal endothelium and the potential toxic effects that can result. Better dyeing can be achieved under air because viscoelastic can block the dye from touching the anterior capsule. However, air bubbles are not stable and anterior chamber loss may occur while introducing the cannula into the anterior chamber for dye injection. A small amount of viscoelastic can be injected through the side port to prevent the air bubble escaping from the anterior chamber. The anterior capsule can also be stained with trypan blue under viscoelastic. Another alternative staining method may be to use a mixture of viscoelastic and trypan blue.

We chose to use a dispersive-cohesive viscoelastic agent in our patients because we anticipated that these may be difficult cases. Due to the greater space-maintaining property of this agent, it is possible it displaced the stain posteriorly. The longer dwell time in the anterior chamber and the stronger coating of the tissues and endothelial protection increased the success of our surgery.

In cataract surgery, it may be necessary to use iris retractors and trypan blue simultaneously. In this case, the retractors should be positioned parallel to the iris plane and iris tenting should be avoided. By injecting trypan blue under viscoelastic, which creates a confined space in the anterior chamber, the posterior migration of the dye under the iris can be prevented. The creation of a viscoelastic barrier to the peripheral iridolenticular space may also prevent posterior migration of dye. However, the desired degree of staining may not be achieved if sufficient contact with the anterior capsule is not ensured.

Retinal toxicity was not observed in our patients. However, it should not be forgotten that trypan blue in high concentrations (above 0.5%) may have toxic effects on the retina.^3,7,15,16^

In conclusion, trypan blue staining of the posterior capsule and anterior vitreous can occur during phacoemulsification in eyes with risk factors related to cataract surgery as well as in eyes with no zonular pathology. Trypan blue in the posterior segment causes no detectable early or late problems other than increasing the risk of surgical complications by interfering with visualization of the posterior capsule and capsulorhexis intraoperatively, and it disappears after the first day.

## Figures and Tables

**Table 1:**
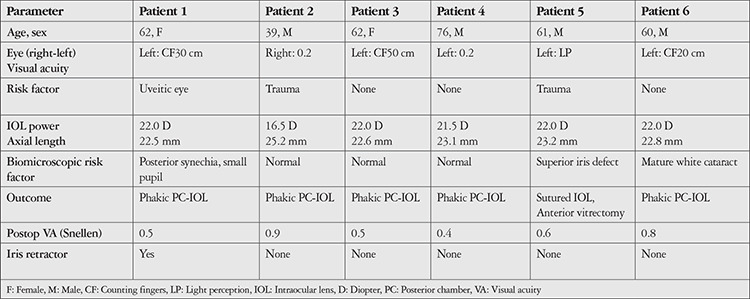
Characteristics of the patients in whom trypan blue used for capsule staining during cataract surgery dyed the anterior vitreous and posterior capsule (the fact that none of the patients used prostate medication was emphasized in the text and therefore was not included in the table). Clearance times could not be determined because the patients were followed up on an outpatient basis and were not hospitalized

## References

[1] Jacobs DS, Cox TA, Wagoner MD, Ariyasu RG, Karp CL;, American Academy of Ophthalmology; Ophthalmic Technology Assessment Committee Anterior Segment Panel (2006). Capsule staining as an adjunct to cataract surgery: a report from the American Academy of Ophthalmology. Ophthalmology..

[2] Sharma N, Bhartiya P, Sinha R, Vajpayee RB (2002). Trypan blue assisted phacoemulsification by residents in training. Clin Exp Ophthalmol..

[3] Bacsal KME, Chee SP (2006). Trypan blue-associated retinal toxicity post complicated cataract surgery. Eye (Lond)..

[4] Chowdhury PK, Raj SM, Vasavada AR (2004). Inadvertent staining of the vitreous with trypan blue. J Cataract Refract Surg..

[5] Gaur A, Kayarkar VV (2005). Inadvertent vitreous staining. J Cataract Refract Surg..

[6] Birchall W, Raynor MK, Turner GS (2001). Inadvertent staining of the posterior lens capsule with trypan blue dye during phacoemulsification. Arch Ophthalmol..

[7] Kheirkhah A, Nazari R, Roohipour R (2010). Inadvertent vitreous staining with trypan blue in pseudoexfoliation syndrome. Arch Ophthalmol..

[8] Marques FF, Soccol FM, Marques DMV, Rehder JRCL (2006). [Unintentional staining of lens posterior capsule with trypan blue during extracapsular cataract extraction: case report]. Arq Bras Oftalmol..

[9] Pelit A (2012). Unintentional staining of the posterior lens capsule with trypan blue dye during phacoemulsification: case report. Int Ophthalmol..

[10] Burkholder BM, Srikumaran D, Nanji A, Lee B, Weinberg RS (2013). Inadvertent trypan blue posterior capsule staining during cataract surgery. Am J Ophthalmol..

[11] Wang BZ, Forward H, Farber L, Chiu D, Chandra A (2014). Surgical blues: vitreous staining with trypan blue during cataract surgery. Clin Exp Ophthalmol..

[12] Marques DMV, Marques FF, Osher RH (2004). Three-step technique for staining the anterior lens capsule with indocyanine green or trypan blue. J Cataract Refract Surg..

[13] Khokhar S, Pangtey MS, Panda A, Sethi HS (2003). Painting technique for staining the anterior lens capsule. J Cataract Refract Surg..

[14] Kayikiçioğlu O, Erakgün T, Güler C (2001). Trypan blue mixed with sodium hyaluronate for capsulorhexis. J Cataract Refract Surg..

[15] Kwok AKH, Yeung CK, Lai TYY, Chan KP, Pang CP (2004). Effects of trypan blue on cell viability and gene expression in human retinal pigment epithelial cells. Br J Ophthalmol..

[16] Rezai KA, Farrokh-Siar L, Gasyna EM, Ernest JT (2004). Trypan blue induces apoptosis in human retinal pigment epithelial cells. Am J Ophthalmol..

